# Vessel Wall MRI in HIV-Associated Cerebral Angiitis

**DOI:** 10.5334/jbsr.2162

**Published:** 2020-11-09

**Authors:** Soufiane Arktout

**Affiliations:** 1 Erasme Hospital, BE

**Keywords:** Angiitis, HIV, Vasculitis, Stroke, MRI

## Abstract

**Teaching Point:** Angiitis, an underestimated cause of stroke in HIV patients, can be diagnosed using vessel wall MRI which directly demonstrates arterial wall thickening and enhancement.

## Case Report

A 58-year-old man presented to the emergency room for confusion over a period of 24 hours. The patient had a history of type 2 diabetes, hypertension, cigarette smoking, and HIV infection, well treated over 12 years with undetectable viral load. The neurologic examination did not show any focalization sign.

**Figure 1:**
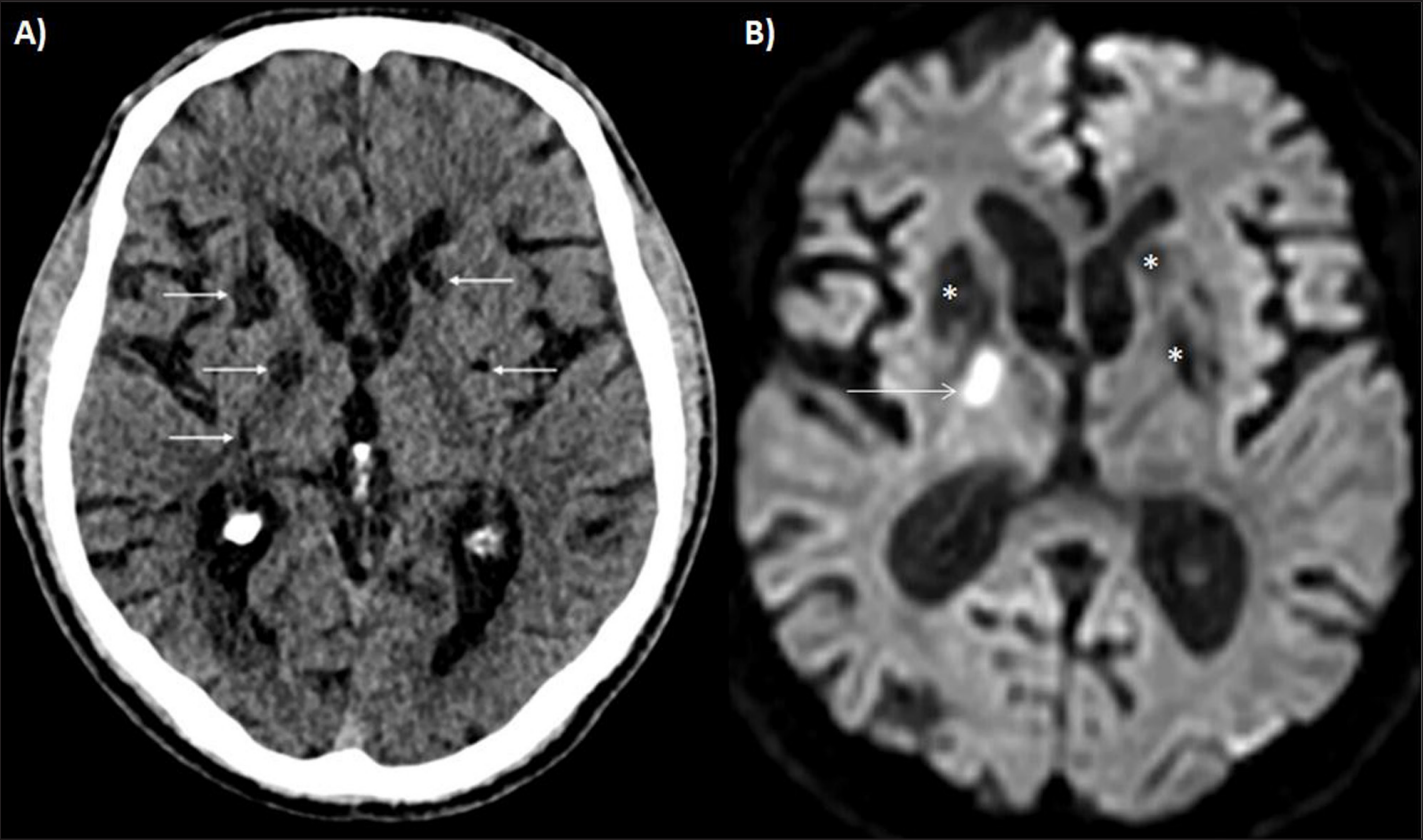


**Figure 2:**
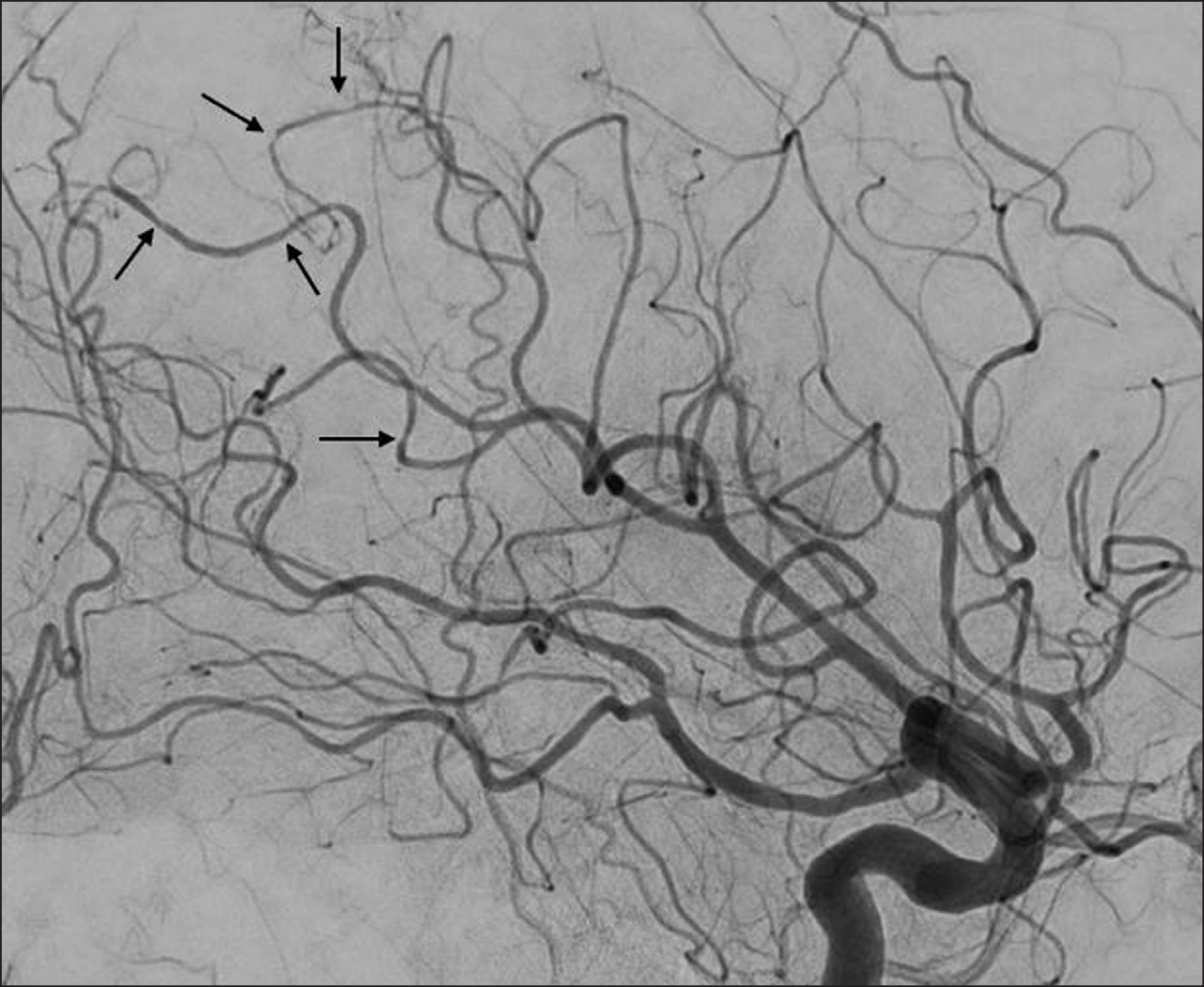


Cerebral computed tomography (CT) demonstrated multiple sequelae in the basal ganglia (arrows on Figure 1A). The cerebrospinal fluid analysis showed an elevated cell count (12/mm^3^ – N <3–5 / mm^3^) and proteins (102 mg/dl – N <30 mg/dl). The patient was hospitalized and a brain magnetic resonance imaging (MRI) showed multiple ischemic lesions of different ages (Figure 1B: diffusion-weighted imaging at the same level as the CT, showing a restriction due to an ischemic event in the right basal ganglia [arrow] and bilateral areas of increased diffusion consistent with sequelae [asterisks]). This atypical pattern of lesions led to complementary investigations. Whilst the rest of the cardio-vascular work-up was normal, cerebral digital subtraction angiography (DSA) demonstrated multiple irregularities and stenosis on the distal arteries (arrows on Figure 2).

**Figure 3:**
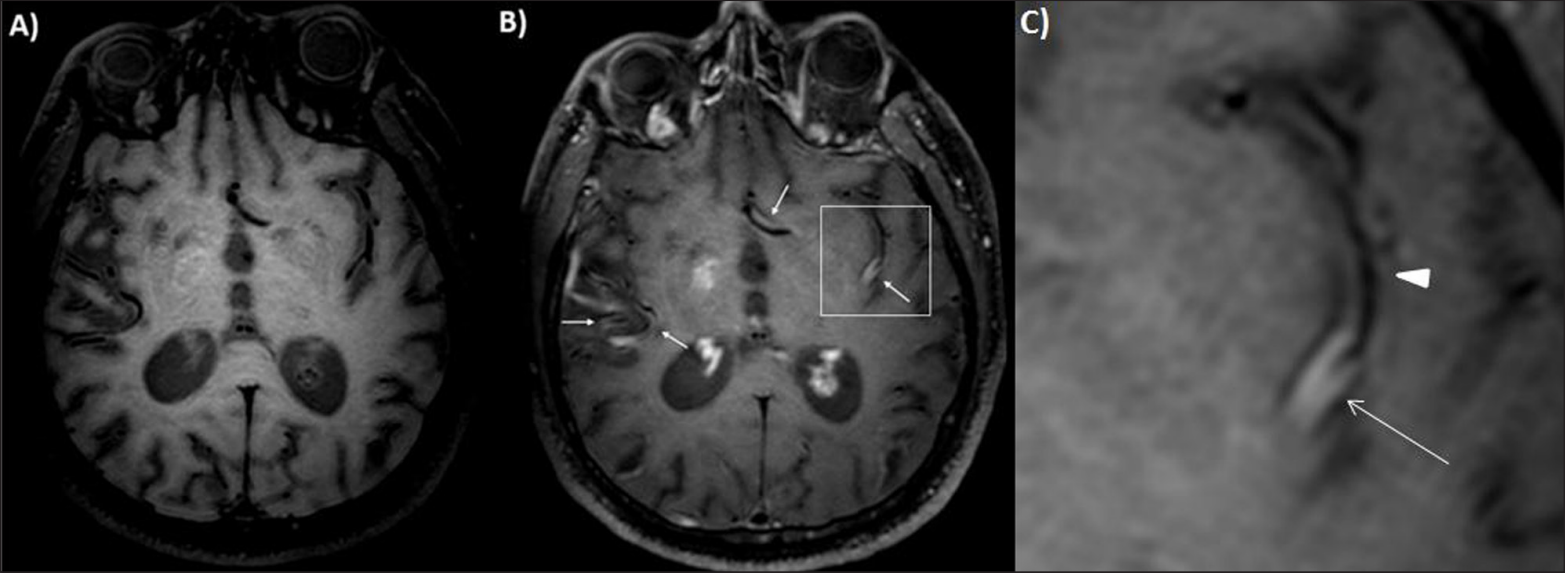


A complementary MRI investigation was performed using a black-blood sequence, which is a high-resolution 3D vessel wall magnetic resonance (VW-MR) technique done before (Figure 3A) and after (Figure 3B) intravenous injection of Gadolinium. It demonstrated multifocal arterial wall thickening and circumferential enhancement on the middle cerebral arteries (arrows on Figure 3B). The Figure 3C magnifies the left middle cerebral artery and clearly shows the thickening and the enhancement of the arterial wall with narrowing of the lumen (arrow) in opposite with the proximal part of the artery which appears normal (arrowhead).

These observations lead to the diagnosis of HIV-associated cerebral angiitis.

The treatment was based on corticosteroid and cyclophosphamide administration. The evolution was favorable with resolution of the symptoms after 10 days.

## Discussion

Stroke in HIV-positive patients is a complex and multifactorial process. It could be related to the HIV infection itself, immunosuppression, or the antiretroviral therapy. In addition, traditional risk factors for vascular disease (hypertension, diabetes, dyslipidaemia, etc.) are more frequent in aging HIV-positive population. The spectrum of etiologies is wide, including large artery atherosclerosis, small vessel disease, cardio-embolism, infection-associated stroke, coagulopathy, and inflammatory vasculopathy (angiitis). Thirteen to 28% of strokes in HIV-positive population are considered to be secondary to angiitis of the central nervous system.

Vessel-wall imaging techniques have seen important steps in their development in the recent years, allowing to assess in vivo the abnormalities of the vessel walls, in opposite to the other angiographic techniques (especially DSA) which demonstrate the repercussions of the arterial wall disease on the lumen. VW-MR imaging typically shows a circumferential thickening and enhancement of the vessel wall in angiitis whereas in atherosclerotic lesions the enhancement is usually eccentric [1].

The treatment of the HIV-associated angiitis of the central nervous system is based on the combination of immunosuppression and anti-viral agents.
